# The role and clinical significance of microRNA-29a-3p in the development of hypopharyngeal carcinoma

**DOI:** 10.1016/j.bjorl.2023.03.001

**Published:** 2023-03-10

**Authors:** Tao Liu, Detao Ding, Wei Wang, Yungang Wu, Dengdian Ma, Miaomiao Liu, Ziqiao Tan, Jing Yao, Xiaoyu Li

**Affiliations:** a Affiliated Hospital of Jining Medical University, Department of Otolaryngology, Head and Neck Surgery, Jining, China; b Affiliated Hospital of Jining Medical University, Department of Pathology, Jining, China; c Public Health College of Jining Medical University, Jining, China; d Basic Medical College of Jining Medical University, Jining, China; e Jining Key Laboratory of Pharmacology, Jining Medical University, Jining, China

**Keywords:** Hypopharyngeal cancer, MicroRNA-29a-3p, Ki67, E-cadherin, Survival time

## Abstract

**Objective:**

MicroRNA-29a-3p has been reported in a variety of cancers, but its role in hypopharyngeal cancer remains unclear. This study was to determine the role of microRNA-29a-3p in the occurrence and development of hypopharyngeal cancer.

**Methods:**

40 patients with hypopharyngeal cancer who underwent surgery in the Affiliated Hospital of Jining Medical University from April 2013 to November 2017 were selected for this study. The cancer tissue samples of the patients were collected, and the patients were followed up for three years. The expression of microRNA-29a-3p in tissue samples was detected by in situ hybridization with fluorescent probe, and the relationships among microRNA-29a-3p and clinicopathological factors, postoperative recurrent-metastasis, survival time were studied. Immunohistochemical was used to detect the expression of Ki67 and E-cadherin in tissue samples.

**Results:**

Combined with HE staining results showed that microRNA-29a-3p expression was relatively high in non-cancer tissue cells (red blood cells and fibroblasts in tumor interstitial vessels), but was relatively low in cancer tissue and cells. According to the follow-up data of 40 patients with hypopharyngeal cancer, tumor size, T-stage, tumor differentiation, postoperative recurrent-metastasis of hypopharyngeal cancer patients were significantly negatively correlated with microRNA-29a-3p (*p* < 0.05). Immunohistochemica results further confirmed that microRNA-29a-3p was negatively correlated with the expression of Ki67 and E-cadherin. The survival time of patients positively related with microRNA-29a-3p expression (*p* < 0.05). Moreover, ROC curve analysis showed that the area under the curve of the combined detection of miRNA-29a-3p+Ki67+E-cadherin was larger than that of the single detection of the three indexes.

**Conclusions:**

The expression of microRNA-29a-3p is closely related to the occurrence, development and prognosis of hypopharyngeal cancer, and it affects the proliferation and invasion. This indicates that microRNA-29a-3p serves as a therapeutic target for the occurrence and development of hypopharyngeal cancer.

The evidence of study designs of this study is IV using “Oxford Centre for Evidence-Based Medicine 2011 Levels of Evidence”.

## Highlights

• The results showed that microRNA-29a-3p expression was relatively high in non-cancer tissue cells but was relatively low in cancer tissue.
• Some clinicopathologic features, the expression of Ki67 and E-cadherin, and the survival time of HPC patients were correlated with miRNA-29a-3p.
• This study found the relationship between miRNA-29a-3p and the occurrence and development of HPC.
• This study will contribute to the individualized treatment of HPC, which improve the treatment strategy and prognosis evaluation system of HPC.

## Introduction

Hypopharyngeal Cancer (HPC) is a common malignant tumor of otorhinolaryngology, which is considered to be one of the head and neck tumor with poor prognosis.^1^, ^2^, ^3^, ^4^ It is a malignant tumor of unknown etiology originating in the root of the tongue, epiglottis, piriform fossa, epiglottis folds of the arytenoid, posterior cricoid area, lateral and posterior wall of the larynx and pharynx, which is clinically dominated by squamous cell carcinoma.^3^, ^4^, ^5^ Since hypopharyngeal cancer is prone to cervical lymph node metastasis and its location is hidden, ipsilateral cervical lymph node metastasis was found in 60%–80% of patients during initial treatment, and contralateral cervical lymph node metastasis was found in 40% of patients.^2^, ^3^, ^4^ At present, hypopharyngeal cancer is mainly treated by surgery, supplemented by perioperative radiotherapy and chemotherapy. Therefore, it is essential to search new HPC-associated biomarkers, improve the accuracy of early HPC diagnosis and reduce the missed diagnosis rate.

MicroRNAs (miRNAs) are a class of small, non-coding, single-stranded endogenous RNAs with 19–25 nucleotides in length, which participate in the regulation of post-transcriptional gene expression in plants and animals, and negatively regulate gene expression through complementary pairing with target mRNA at the transcriptional level, resulting in inhibition or degradation of mRNA translation. Existing findings have proved that miRNAs play an important role in eukaryotic gene regulation and are widely involved in various physiological activities such as cell proliferation, differentiation, development, metabolism and apoptosis.^6^, ^7^, ^8^ MiRNA expression is significantly different in normal tissues and tumor tissues, and is involved in the occurrence and development of tumors.^7^, ^8^, ^9^

It has been reported that miRNA expression was low in tumor tissues compared with normal tissues, which is closely related to tumor occurrence, invasion and metastasis in a variety of tumors, including gastric cancer, ovarian cancer, liver cancer, glioma, breast cancer, prostate cancer, colorectal cancer, nasopharyngeal carcinoma, thyroid papillary carcinoma, renal clear cell carcinoma, laryngeal cancer, and cervical cancer.^10^, ^11^, ^12^, ^13^, ^14^, ^15^, ^16^, ^17^, ^18^, ^19^, ^20^, ^21^, ^22^, ^23^, ^24^, ^25^, ^26^, ^27^, ^28^, ^29^, ^30^ However, the effect of miRNA-29a-3p on the occurrence and development of HPC has not been reported at home or abroad. This study aims to explore the role of miRNA-29a-3p in occurrence and development of HPC, which is conducive to the early diagnosis and prognosis evaluation of tumor and provides a promising research direction for exploring the occurrence, invasion, and metastasis mechanism of HPC, as well as searching for new diagnostic markers and therapeutic targets. The clinicopathological information, survival time, postoperative recurrence, and metastasis of 40 HPC patients were collected to screen potential markers for poor prognosis of HPC.

## Methods

### Patients

A total of 40 patients admitted to the Department of Otolaryngology, Head and Neck Surgery, Affiliated Hospital of Jining Medical University from April 2013 to November 2017 were collected. All patients were diagnosed with HPC by pathological biopsy and treated with surgery, aged 42–75 years.

The clinical staging method was based on the guidelines for the diagnosis and treatment of head and neck tumors of Chinese society of clinical oncology (2021).

All patients were followed up for more than 36 months.

Patients were selected for strict inclusion criteria: patients pathologically diagnosed with HPC without distant metastasis and who agreed to surgical treatment.

The inclusion criteria were strictly followed in this study, and all patients were pathologically diagnosed with HPC without distant metastasis and were treated with surgery.

### The main reagents

The fluorescence in situ hybridization probe was purchased from Thermo Fisher Scientific (sequence 5’-FAM-TAACCGATTTCAGATGGTGCTA-FAM-3’). Bovine Serum Albumin (BSA), protease-K, Diamamidine Phenylindole (DAPI), antifade solution and hybridization buffer were all purchased from Wuhan Service Biotechnology Co., Ltd. Ki67 and E-cadherin antibodies were purchased from Affinity Biosciences.

### Fluorescence in situ hybridization

Tissue fixation was performed first: the tissue was removed, cleaned, immediately placed in fixative solution more than 12 h, and then dewatered. After the fixation, the tissue was dehydrated by gradient alcohol, dipped in wax, embedded. The paraffin was sliced and baked at 62 °C for 2 h. The slices were placed in xylene ⅰ for 15 min, xylene ⅱ for 15 min, anhydrous ethanol ⅰ for 5 min, finally anhydrous ethanol ⅱ for 5 min, and dried naturally and soaked in diethylpyrocarbonate-treated water. Then boiled in the repair solution for 10 min, cooled naturally, added protease K (20 μg/mL) at 37 °C for 20 min, washed with phosphate buffer saline 3-times, dripped with the pre-hybridization solution and incubated at 37 °C for 1 h, the hybridization solution containing 1 μM miRNA-29a-3p probe overnight at 42 °C. And the slices were dumped the pre-hybridization solution, washed by Saline Sodium Citrate (SSC) at 37 °C for 10 min, 1 × SSC at 37 °C for 2-times, 0.5 × SSC at room temperature for 10 min. The slices were dripped with DAPI dye solution, incubated away from light for 8 min, sealed with antifade solution. Finally, the slices were observed under fluorescence microscope and images were collected (UV excitation wavelength 330–380 nm, emission wavelength 420 nm, blue light; FAM [488] green excitation wavelength 465–495 nm, emission wavelength 515–555 nm, green light; CY3 excitation wavelength 510–560 nm, emission wavelength 590 nm, red).

### Immunohistochemistry (IHC)

Expression of Ki67 and E-cadherin in colonic tissue sections was assessed by IHC as previously described.^31^

The IHC results were quantified, and positive scores were assessed by the degree of staining in each patient's tissue sections. The mean positive intensity of the measurement area is 0, 1, 2, 3 points: negative without staining, 0-points; weak positive light yellow, 1-point; medium positive brownish yellow color, 2-points; strong positive tan count 3-points. The positive rate of cells was rated as 0-points for 0%–5%, 1-point for 6%–25%, 2-points for 26%–50%, 3-points for 51%–75%, and 4-points for >75%. The positive comprehensive score was staining intensity multiply by positive cell ratio score. The expression levels were determined by averaging the positive scores for six fields in tissue sample.

### Statistical analysis

We used X-tile software to define the expression level of miRNA-29a-3p, divided the samples into high expression groups and low expression groups, and selected 0.027955 as the cut-off value.

Qualitative data were described by frequency table. Statistical inference of qualitative data in Table 1 was carried out by Chi-Square test or Fisher’s exact probability method, and *p* < 0.05 was considered statistically significant. Spearman method was used for correlation analysis of two variables in Table 2, Table 3, *p* < 0.05 and *p* < 0.01 were considered statistically significant. Including the Log-rank test in Kaplan-Meier survival analysis, *p* < 0.05 was considered statistically significant. The survival time ranged from the date of surgery to the date of death or to the time of follow-up.

**Table 1:** Correlation between the expression of miRNA-29a-3p and clinicopathological characteristics in HPC.

Index	miRNA-29a-3p		*p*-value
	High expression (n = 35)	Low expression (n = 5)	
**Age**			> 0.05
< 65 (n = 23)	21	2	
≥ 65 (n = 17)	14	3	
**Smoking**			> 0.05
No (n = 9)	8	1	
Yes (n = 31)	27	4	
**Drinking**			> 0.05
No (n = 19)	16	3	
Yes (n = 21)	19	2	
**Tumor size**			0.030^a^
< 4 cm (n = 33)	31	2	
≥ 4 cm (n = 7)	4	3	
**Tumor differentiation**			0.009^b^
Poor differentiation (n = 6)	3	3	
Moderate differentiation (n = 25)	23	2	
High differentiation (n = 9)	9	0	
**T-stage**			0.046^a^
T1‒T3 (n = 32)	30	2	
T4 (n = 8)	5	3	
**N-stage**			> 0.05
N0‒1 (n = 13)	11	2	
N2‒3 (n = 27)	24	3	
**Tumor site**			> 0.05
The piriform fossa type (n = 35)	30	5	
Other area (n = 5)	5	0	
**Postoperative recurrent metastasis**			0.013^a^
No (n = 22)	22	0	
Yes (n = 18)	13	5	

a *p* < 0.05.

b *p* < 0.01.

**Table 2:** Correlations among the expression of miRNA-29a-3p, Ki67 and E-cadherin.

Index	n	Correlation index	*p*-value
Ki67	40	−0.317	0.046^a^
E-cadherin	40	−0.429	0.006^b^

Spearman method was used for correlation analysis of two variables.

a *p* < 0.05.

b *p* < 0.01.

**Table 3:** Correlation between the survival time and the expression of miRNA-29a-3p.

Index	n	Correlation index	*p*-value
miRNA-29a-3p	40	0.388	0.013^a^

Spearman method was used for correlation analysis of two variables.

a *p* < 0.05.

## Results

### Expression of miRNA-29a-3p in HPC tissue samples

**Figure 1:**
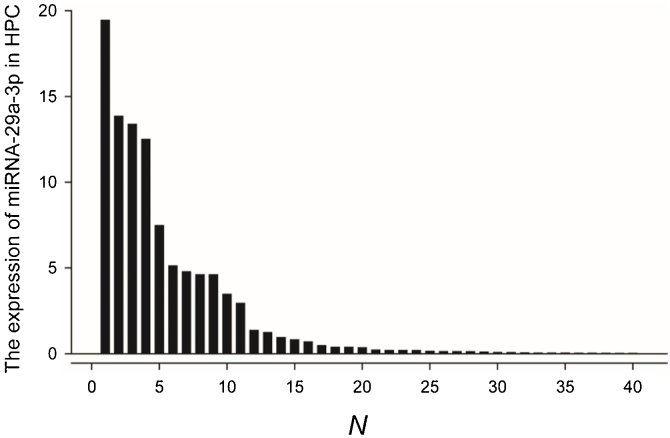
Expression of miRNA-29a-3p in hypopharyngeal cancer tissue samples.

Fluorescence probe in situ hybridization was used to detect miRNA-29a-3p expression in tissue sample from 40 patients with HPC, and the expression levels were quantified as shown in Fig. 1.

### The comparation of miRNA-29a-3p expression in HPC tissues and paracancer tissues

**Figure 2:**
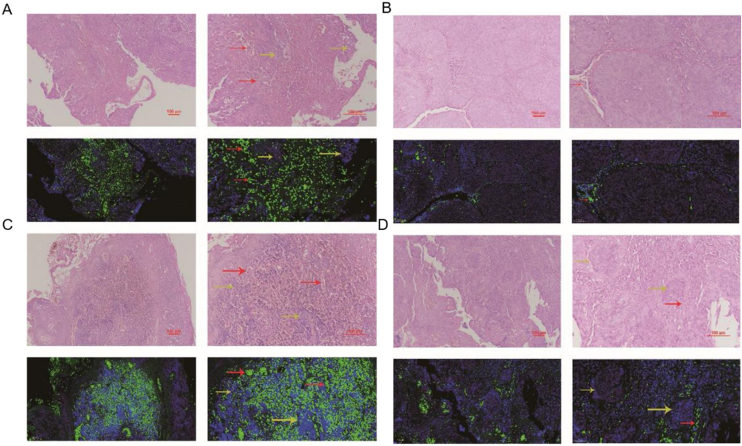
The expression miRNA-29a-3p in hypopharyngeal cancer and adjacent normal tissues (Patient 1#). Red arrows point to tumor interstitial vascular red blood cells, and yellow arrows point to tumor cells.

**Figure 3:**
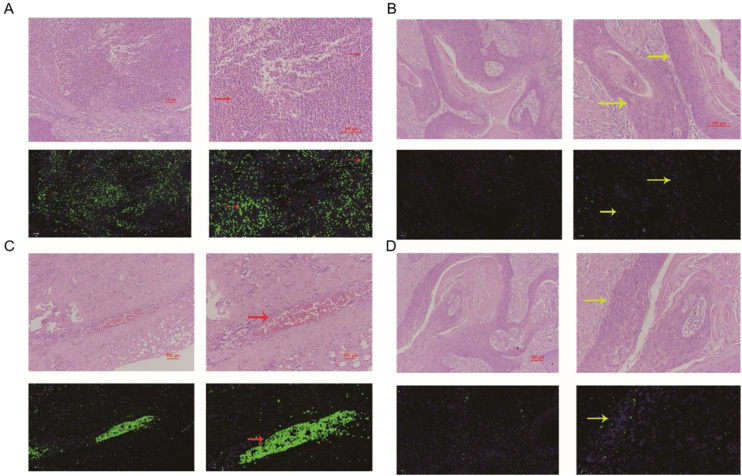
miRNA-29a-3p expression in hypopharyngeal cancer and adjacent normal tissues (Patient 2#). Red arrows point to tumor interstitial vascular red blood cells, and yellow arrows point to tumor cells.

**Figure 4:**
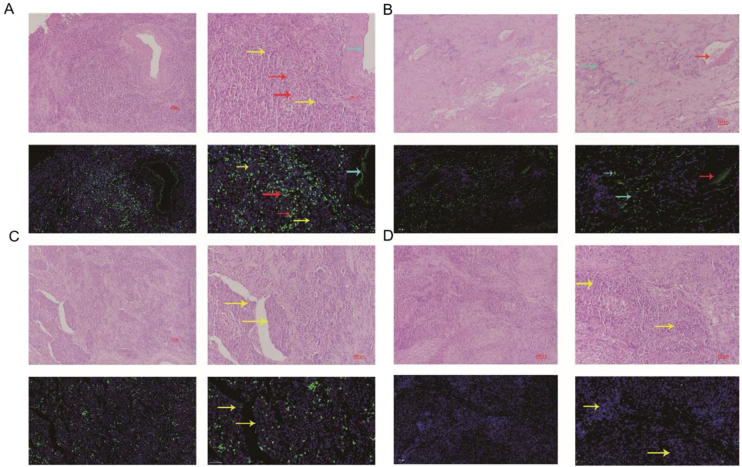
miRNA-29a-3p expression in HPC and adjacent normal tissues (Patient 3#). Red arrows point to tumor interstitial vascular red blood cells, light blue arrows point to fibroblasts, and yellow arrows point to tumor cells.

The expression of miRNA-29a-3p was detected by fluorescence probe in situ hybridization. We selected tissue samples from three cancer patients with high miRNA-29a-3p expression. As shown as Fig. 2, Fig. 3 and Fig. 4, the expression of miRNA-29a-3p was mainly located in the cytoplasm. Combined with HE is staining results, miRNA-29a-3p expression level was relatively high in non-cancer tissues or cells (including red blood cells and fibroblasts), but low in cancer tissues.

### The association between the expression of miRNA-29a-3p and clinicopathological features in HPC

The relationship between the expression of miRNA-29a-3p and clinicopathological features in HPC patients are summarized in Table 1. A significant correlation was observed between the expression of miRNA-29a-3p and tumor size (*p* < 0.05). The increased expression of miRNA-29a-3p were related to a decrease in tumor cell differentiation (*p* < 0.01). Moreover, the expression of miRNA-29a-3p was negatively correlated with the T-stage (*p* < 0.05). MiRNA-29a-3p expression levels in HPC patients with relapse and metastasis were significantly lower than that observed in HPC patients without relapse and metastasis (*p* < 0.05). However, there was no significant correlation between the expression of miRNA-29a-3p and age (*p* = 0.634), smoking (*p* = 0.783), drinking (*p* = 0.654), N-stage (*p* > 0.05) or tumor site (*p* > 0.05).

### The association among the expression of miRNA-29a-3p, Ki67and E-cadherin in HPC tissues

The relationship among the expression of miRNA-29a-3p, Ki67 and E-cadherin in HPC patients is summarized in Table 2. The results showed that there were significant correlations between the expression of miRNA-29a-3p and Ki67 (*p* < 0.05), or E-cadherin (*p* < 0.05).

### The association between the expression of miRNA-29a-3p and the survival time of HPC

**Figure 5:**
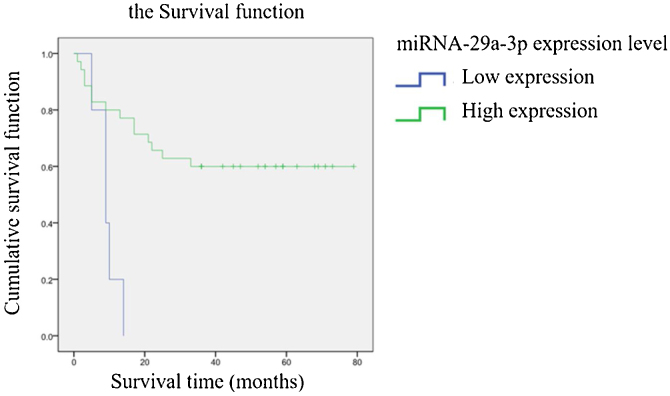
The survival time of patients with high and low miRNA-29a-3p expression.

The relationship between the expression of miRNA-29a-3p and survival time in HPC patients is summarized in Table 3. A significant correlation was observed between the expression of miRNA-29a-3p and survival time (*p* < 0.05). Kaplan-Meier survival analysis showed that the survival time of patients with high and low miRNA-29a-3p expression was significantly different (Log Rank Chi-square value = 10.501, *p* < 0.01), and the results are shown in Fig. 5. These results showed that survival time of HPC patients with high miRNA-29a-3p expression was significantly increased.

### The role of combined detection of Ki67, E-cadherin and miRNA-29a-3p in the prognosis of hypopharyngeal cancer

**Figure 6:**
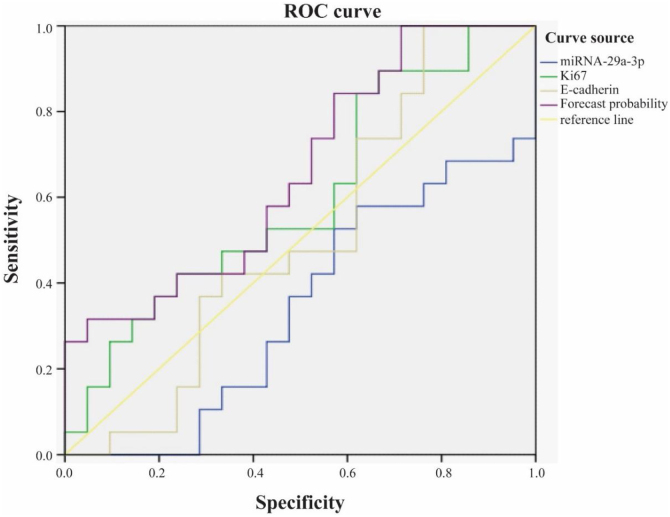
The role of combined detection of Ki67, E-cadherin and miRNA-29a-3p in the prognosis of hypopharyngeal cancer.

We used ROC curve to analyze the role of combined detection of Ki67, E-cadherin and miRNA-29a-3p in the prognosis of hypopharyngeal cancer (Fig. 6). The results showed that the area under ROC curve for survival of patients with hypopharyngeal cancer by Ki67 alone was 0.599, and the 95% Confidence Interval (CI) was 0.421∼0.777. The area under ROC curve by E-cadherin alone was 0.509, and the 95% CI was 0.325∼0.693. Nevertheless, the area under ROC curve of combined detection of miRNA-29a-3p+Ki67+E-cadherin was 0.659, and 95% CI was 0.49∼0.829. That is to say, the area under the curve of the combined detection of the three indexes was larger than that of the single detection of the three indexes.

## Discussion

The current clinicopathological staging method has limitations in evaluating the prognosis of HPC patients. It is essential to explore anew molecular marker to make prognosis evaluation more reliable and accurate. Our results demonstrated that the miRNA-29a-3p levels in HPC patients were widely associated with clinicopathological characteristics representing tumor cell progression and poor prognosis, and it maybe a potential tumor marker to evaluate the prognosis of hypopharyngeal carcinoma after surgery.

In this study, fluorescence in situ hybridization experiment and HE staining experiment showed that miRNA-29a-3p was highly expressed in non-cancer tissues or cells, mainly in red blood cells or connective tissues, and relatively low in cancer tissues. This result is consistent with the expression trend of miRNA-29a-3p in other types of cancer reported in previous literature.^10^, ^11^, ^12^, ^13^, ^14^, ^15^, ^16^, ^17^, ^18^, ^19^, ^20^, ^21^, ^22^, ^23^, ^24^, ^25^, ^26^, ^27^, ^28^, ^29^, ^30^ Combined with the clinical data of patients, the results showed that the expression level of miRNA-29a-3p was negative correlated with tumor size, tumor ‒ stage, tumor differentiation, postoperative recurrent-metastasis and survival time of HPC patients (*p* < 0.05) as shown as Table 2. That is to say, HPC patients with low expression of miRNA-29a-3p had more tumors with a diameter greater than or equal to 4 cm, poor differentiation, and these patients are more prone to postoperative recurrence and metastasis. Survival analysis found that the survival time of HPC patients with high miRNA-29a-3p expression was significantly increased and the prognosis was better. However, there was no significant correlation between the expression of miRNA-29a-3p and age (*p* = 0.634), smoking (*p* = 0.783), drinking (*p* = 0.654), N-stage (*p* > 0.05) or tumor site (*p* > 0.05).

It has been reported that miRNA-29a-3p is closely related to the tumor occurrence and development through various mechanisms.^10^, ^11^, ^12^, ^13^, ^14^, ^15^, ^16^, ^17^, ^18^, ^19^, ^20^, ^21^, ^22^, ^23^, ^24^, ^25^, ^26^, ^27^, ^28^, ^29^, ^30^ MiRNA-29a-3p inhibited the proliferation, invasion and metastasis of gastric cancer cells, and the expression level of miRNA-29a-3p in gastric cancer tissue samples was significantly lower than that in adjacent normal tissue samples.^10^, ^25^ Inhibition of miRNA-29a-3p expression promotes epithelial mesenchymal transformation, and long-chain non-coding RNA (lncRNA) DUXAP8 was negatively correlated with miRNA-29a-3p expression in ovarian cancer tissues.^11^, ^12^, ^13^ MiRNA-29a-3p has a low expression level in liver cancer tissue, plays an important role in tumorigenesis, invasion and metastasis, and down-regulates hepatocellular carcinoma prognosis.^14^, ^15^, ^16^, ^17^, ^18^ MiRNA-29a-3p inhibits glioma cell proliferation, migration and angiogenic mimicry formation, and further studies confirm that roundabout1 is a direct target of miRNA-29a-3p.^19^, ^20^ MiRNA-29a-3p significantly inhibited the proliferation, invasion and migration of breast cancer cells and promoted apoptosis.^21^ MiRNA-29a-3p inhibits the proliferation and invasion of prostate cancer cells and induces apoptosis.^22^ MiRNA-29a-3p attenuates colorectal cancer cell proliferation by targeting down ribosomal S15A.^23^ The expression of miRNA-29a is low in radio-resistant nasopharyngeal carcinoma tissues, and miRNA-29a induces the radio-sensitivity of tumor cells by inhibiting cell viability and promoting cell apoptosis.^24^ MiRNA-29a-3p inhibits the growth, proliferation and invasion of thyroid papillary carcinoma by inhibiting the NF-kappaB signaling pathway.^26^ Compared with non-cancerous tissues, miRNA-29a-3p expression in renal clear cell carcinoma was significantly reduced.^27^ MiRNA-29a-3p targets downregulation of Prominin1 in laryngeal cancer tissues and cells to inhibit tumor proliferation.^28^, ^29^ MiRNA-29a-3p directly targets Smad nuclear reactive protein 1 and inhibits the migration and proliferation of cervical cancer cells.^30^ It is reported that patients with high Ki67 and E-cadherin positive rate had faster tumor growth, worse tissue differentiation and worse prognosis.^32^, ^33^, ^34^ The results of IHC experiments found that miRNA-29a-3p was significantly negatively correlated with the expression of Ki67 and E-cadherin (*p* < 0.05), that is, the high expression of miRNA-29a-3p inhibited the proliferation, invasion and metastasis of HPC cells. Kaplan-Meier survival analysis showed that survival time of HPC patients with high miRNA-29a-3p expression was significantly increased as shown as Fig. 5 and Table 3. Moreover, as shown as Fig. 6, ROC curve analysis showed that combined detection of miRNA-29a-3p+Ki67+E-cadherin had the highest value for the prognosis of hypopharyngeal cancer, and the judgment was more accurate, which could provide good clinical guidance.

It's worth noting that the molecular mechanism of miRNA-29a-3p action on the proliferation, invasion, and metastasis of HPC remains unclear, and it needs to be further studied *in vivo* and *in vitro*. We will use the simulators and inhibitors of miRNA-29a-3p transfected into HPC cell lines to explore the effects and mechanism of miRNA-29a-3p on tumor proliferation, invasion, and metastasis. Moreover, miRNA-29a-3p-related signaling pathways will be further studied by using protein chip and other technologies.

## Conclusions

This study for the first time found the relationship between miRNA-29a-3p and the occurrence and development of HPC, and miRNA-29a-3p maybe a valuable prognostic indicator of HPC. This study will contribute to the individualized treatment of HPC, which improve the treatment strategy and prognosis evaluation system of HPC.

## Author's contribution

Xiaoyu Li, Tao Liu, Detao Ding, Yungang Wu, Dengdian Ma, Ziqiao Tan work at Department of Otolaryngology, Head and Neck Surgery, Affiliated Hospital of Jining Medical University. All operations for hypopharyngeal cancer in this study were performed by the above authors.

Jing Yao works at Jining Medical University. The molecular experiments, such as immunohistochemistry and hematoxylin and eosin staining, were carried out by Jing Yao and Tao Liu.

Wei Wang works at Department of Pathology, Affiliated Hospital of Jining Medical University. All pathological sections used in this paper were completed by Dr. Wei Wang.

Miaomiao Liu works at Jining Medical University. All the statistical analyses in the paper were completed under the guidance of Dr. Liu Miaomiao.

## Funding

This work was supported by the grants from the National Natural Science Foundation of China (nº 81603143), Natural Science Foundation of Shandong Province (nº ZR2019MH059); Shandong Traditional Chinese Medicine Science and Technology Project (nº 2020Q060; M-2022247); Research Fund for Lin He’s Academician Workstation of New Medicine and Clinical Translation in Jining Medical University (nº JYHL2021MS30; JYHL2021FMS13); Practical Teaching Education Research Project of Jining Medical University (nº JYSJ2022B44); Scientific research and Innovation Team of Jining Medical University (Cultivation): Digestive System Inflammation and Tumor Molecular Pharmacology Research Innovation Team.

## Conflicts of interest

The authors declare no conflicts of interest.
